# Major decrease in malaria transmission on Mayotte Island

**DOI:** 10.1186/s12936-015-0837-6

**Published:** 2015-08-19

**Authors:** Olivier Maillard, Tinne Lernout, Sophie Olivier, Aboubacar Achirafi, Lydéric Aubert, Jean François Lepère, Julien Thiria, Frédéric Pagès, Laurent Filleul

**Affiliations:** Regional Office of the French Institute for Public Health Surveillance (InVS), Mayotte, France; Epidemiology of Infectious Diseases, Scientific Institute of Public Health, Brussels, Belgium; Hospital Centre of Mayotte, Mayotte, France; Regional Health Agency (ARS), Mayotte, France; Regional Office of the French Institute for Public Health Surveillance (InVS), Ile De France, France

**Keywords:** Malaria, Elimination, *Plasmodium falciparum*, Mayotte, Comoros, Indian ocean

## Abstract

**Background:**

*Plasmodium falciparum* is responsible for most malaria cases on Mayotte Island, in the Comorian Archipelago. Malaria is endemic and a major public health problem in the archipelago with an intense, stable and permanent transmission. This study reports results of 8 years of malaria surveillance from 2007 to 2014 after the strengthening of malaria control activities in Mayotte and the neighbouring islands.

**Methods:**

Surveillance was based on physicians’ reports of malaria cases between January 2007 and December 2014. Malaria cases were confirmed by at least a positive rapid diagnostic test and/or demonstration of *Plasmodium sp*. in a blood smear. The date, and the patients’ age, sex, address, presentation of symptoms, biology, treatment and recent history of travel were collected by verbal questioning during consultation and/or hospitalization. Monthly rainfall data were also compiled during the study period.

**Results:**

From 2007 to 2014, 2073 cases were reported on Mayotte Island: 977 imported cases, 807 autochthonous cases and 289 cases of unknown origin. The total malaria annual parasite incidence lowered from 3.0 in 2007 to 0.07 per 1,000 inhabitants in 2014 as the autochthonous malaria incidence decreased from 1.6 to 0.004 per 1,000 inhabitants in the same period and in all age groups. Most of the imported cases came from Comoros (94 %). Severe forms represented approximately 11 % of cases, and only two deaths have been recorded among the imported cases. Approximately 19 % of cases were hospitalized (3 % in an intensive care unit). There is clearly a decrease in malaria transmission in Mayotte since 2007 and the goal of elimination seems more achievable than ever. In 2011, Mayotte entered the elimination phase when *P. falciparum* API passed under 1 case per 1,000 people at risk.

**Conclusions:**

The combination of vector control measures, active surveillance and case management, including effective treatment with artemisinin-based combination therapy, has been essential to achieve a present status of low and decreasing malaria transmission on the island. Mayotte has entered the elimination phase, but some goals remain to be accomplished before a programme re-orientation toward malaria elimination is contemplated. Moreover, a regional management policy is crucial because this would allow control measures to be targeted and based on a regional surveillance-response system rather than isolated.

## Background

**Fig. 1:**
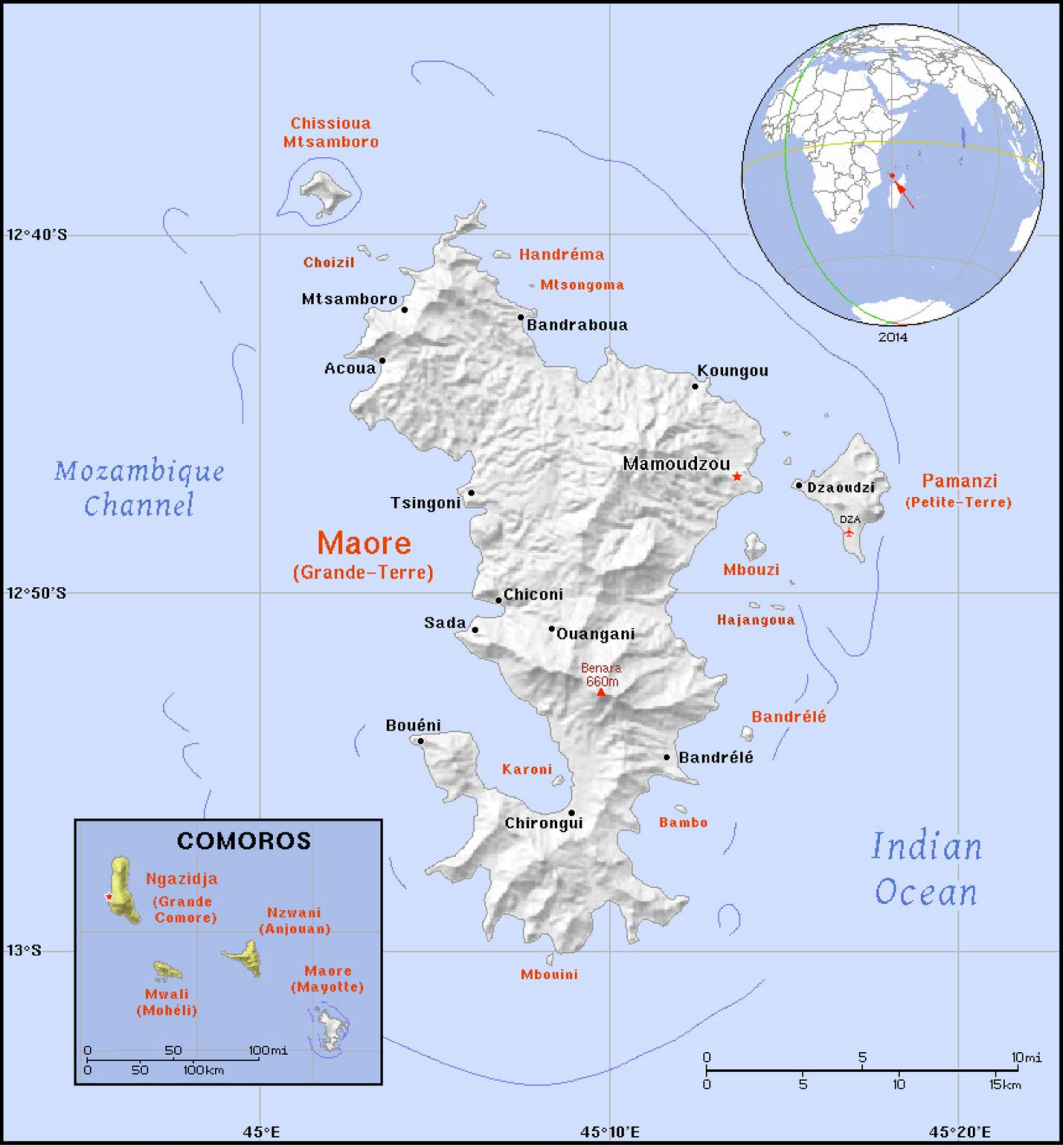
Map and location of Mayotte

Mayotte is a French island located in the Comorian Archipelago in the Indian Ocean between Madagascar and the eastern coast of Africa (Fig. 1). Its population size reached 212,645 inhabitants in 2012 [1], with an annual growth rate of 2.7 % between 2007 and 2012. Legal and illegal immigration is important, and 40 % of inhabitants were of foreign origin in the 2012 census. The climate is tropical and wet with a hot and rainy season, usually from November to April/May and a dry season from May to October. The other three islands of the Comorian Archipelago (Grand Comoro, Anjouan and Moheli) formed the Federal Islamic Republic of Comoros, now called the Union of Comoros, at independence from France in 1975. Malaria is endemic and a major public health problem in the archipelago with an intense, stable and permanent transmission [2].

Eight species of Anopheles mosquitoes have been reported in Mayotte including four species considered to be primary vectors of malaria: *Anopheles gambiae s.s.*, *Anopheles funestus, Anopheles mascarensis* and *Anopheles merus.* The roles of *An. mascarensis* and *An. merus* are not documented but probably minor [3]. Thus, the two main malaria vectors are *An. gambiae s.s.*, permanently distributed all over the island, and *An. funestus.* The two species appeared to be endophilic [4]. The ecology and repartition of *An. funestus* is less well described in Mayotte, but adults were found in Bandraboua, a district in the north of the island where malaria reemerged in 2004 [5].

For many years in Mayotte, the only malaria parasite identified among clinical cases was *Plasmodiumfalciparum* [6]; however, autochthonous cases of *Plasmodium vivax* have been found recently [7]. *Plasmodium ovale* and *Plasmodium malariae* have been reported mainly in the neighbouring islands [8, 9].

Before the 1970s, the malaria control policy was limited in Mayotte. In 1972, the parasite prevalence was 36.5 % among children living on Petite Terre, and a chloroquine prophylaxis program was launched, targeting pregnant women and children under 15 years of age [6].

In 1976, a malaria control unit was created and indoor residual spraying (IRS) and larviciding were initiated. Thus, the overall parasite prevalence decreased from 25.5 % with 511 cases in 1976 to 0.9 % with only 45 cases in 1980 [6, 10], and the goal of elimination was almost achieved [11].

However in 1984, 1987, 1991 and 1995, Mayotte experienced malaria epidemics, and parasite prevalence increased to 2 % in 1996 due in part to decreased control activities at the beginning of the 1980s [12]. Moreover, the use of chloroquine for chemoprophylaxis was stopped in 1986 except for pregnant women because of a reduced efficacy in the Indian Ocean area and according to the World Health Organization (WHO) recommendations at this time [13]. By 2001, the annual parasite incidence (API) was two per 1,000 inhabitants representing approximately 3,000 annual cases. Therefore, according to the Roll Back Malaria recommendations, new malaria control measures were engaged: free distribution of insecticide-treated nets (ITN), especially for newborns and pregnant women, combined in some areas with indoor residual spraying (IRS) with deltamethrin and extensive use of rapid diagnostic tests (RDTs). From 2002 to 2007, patients with uncomplicated malaria received an association of chloroquine and sulfadoxine-pyrimethamine. Three-day regimen artemisinin-based combination therapy (ACT) was then introduced in May 2007 as first-line treatment. At the same time, active and passive surveillance were also strengthened [14]. In 2006, the estimated annual incidence was 3.1 per 1,000 inhabitants with 496 cases clearly on the decline compared with previous reports, especially among children under 5 years of age [7, 14].

This study reports the results of 8 years of malaria surveillance during the period 2007–2014 after the strengthening of malaria control activities in not only Mayotte but also at the regional level in the neighbouring islands. The causes of the decrease of malaria transmission in Mayotte are discussed as the future of malaria control to maintain this low incidence in the regional context.

## Methods

### Study period

All the malaria cases reported in Mayotte from January 2007 to December 2014 have been included retrospectively.

### Epidemiological data

In Mayotte, the malaria case report involves compulsory but passive notifications of cases to the Regional Health Agency (ARS) delegation located on the island. For each case, health practitioners must complete a clinical notification form with case details. The date and the patient’s age, sex, address, presentation of symptoms, biology, treatment and recent history of travel were collected by verbal questioning during consultation and/or hospitalization. Then, related information was collected during the case investigation. RDTs are not used in the field for either symptomatic or asymptomatic people. Only symptomatic people are asked to seek medical advice, including RDT at the nearest public dispensary. After anonymization and validation, data are transmitted to the National Institute for Public Health Surveillance (InVS) for analysis. Surveillance data collection is approved by the French National Commission on Computer Technology and Freedom (CNIL), according to the French regulation on medical confidentiality for mandatory reporting (Authorization no 02-082 on November 19, 2002).

### Case definition

A confirmed malaria case needs laboratory confirmation of infection with or without clinical illness by at least a positive RDT and/or demonstration of *Plasmodium sp*. in a blood smear.

Autochthonous cases are confirmed cases of malaria acquired by mosquito transmission within Mayotte, namely for patients without travel history in the last month for *P. falciparum*.

Imported cases are acquired outside the island with a travel history during the last 30 days for *P. falciparum*.

### Malaria diagnosis

The Hospital Centre of Mayotte is composed of one central hospital, with five peripheral units, and coordinates 13 dispensaries over the island. There is one single public laboratory located in the central hospital and one single private laboratory. Since September 2001, diagnosis can be made at the dispensary level using RDT (OptiMAL IT^®^, Bio Rad Laboratories, France). Blood samples are monitored on the island in the hospital laboratory by trained biologists, and RDT are confirmed by a thin/thick blood smear examination.

### Malaria treatment policy

As the health insurance system has evolved since 2005, dispensaries are still free of charge in Mayotte, so most of the population can consult a health practitioner without having to pay. Nevertheless, people without health insurance, including foreign residents, have to pay a standard amount of 10 Euros for medical advice that includes medical and biological examinations, treatment and hospital admission, if required. The detection of malaria by RDT is mandatory for any consultant with a fever over 38.5 °C. Temperature is usually taken from the ear with a tympanic digital thermometer. Patients found positive for malaria infection by RDT and/or blood smear examination are given anti-malarial treatment depending on clinical and biological criteria. Three-day regimen artemisinin-based combination therapy (ACT) was used as first-line treatment for mild or uncomplicated form. If parasitaemia persists, a second-line treatment is recommended usually by a combination of atovaquone and proguanil, or mefloquine. Severe cases and pregnant women are hospitalized and treated with quinine.

### Vector control

The vector control department (LAV), a specific unit of ARS, was formally created in 2002 when an entomologist was recruited. This unit is devoted to vector control and historically to Anopheles control. It is routinely assigned to the monitoring of the vector populations (adult and larval sampling), larval control, social mobilization, long-lasting insecticidal nets (LLINs) distribution, IRS, space spraying and the assessment of the insecticide resistance of Anopheles vectors, according to standard WHO susceptibility tests. Larval control was made using *Bacillus thuringiensis israelensis* (Bti strain AM65-52, VectoBac WG 37.4 %), temephos or diflubenzuron, a growth inhibitor (IGR). IRS or spatial spraying used deltamethrin as adulticide. From 2007 to 2009, IRS was systematically proposed in all houses over the island with at least one treatment per year. Iterative treatments were also applied to cases’ houses and their neighbourhoods. In the active foci of the island, the objective was to achieve two treatments per year. Since 2010, IRS has been progressively substituted by the distribution of LLINs. LLINs were first proposed to all mothers for newborns in maternities. Then, a systematic distribution of LLINs in malaria hotspots began in 2010 in the active focus of Bandraboua. And the following year, a mass distribution was implemented on the island. Agents of the LAV have installed one LLIN per bed in each house. Currently, 60,000 LLINs have been distributed and installed by the LAV, and only seven villages have not been equipped. IRS was also ongoing in these villages in 2012 and 2013. In 2011, no larval resistance was recorded for Bti or IGR but some populations of *An. gambiae* larvae presented a moderate resistance to temephos [4]. In the same period, all the populations of adult *An. gambiae* were susceptible to deltamethrin.

Furthermore, those routine vector control activities were reinforced by specific field interventions around imported and autochthonous cases. For each malaria case detected by specific epidemiological surveillance, a case investigation called “environmental study” was conducted by personnel of the vector control department to identify the origin of the contamination (house, temporary residence during farming activities or during periods spent in villages named “Tobé”, infection outside of Mayotte), to determine the susceptibility of the area to malaria, to look for vector presence (larval and adult sampling), and to look for other potential malaria cases in the neighbourhood. Then, information about malaria was provided to the local population in the vernacular and LLINs were routinely and freely distributed to the affected and surrounding households. Breeding sites were treated and secondary IRS and space spraying could be proposed.

### Climate data

Temperature and rain data were compiled and supplied by Meteo France from 7 areas at different heights over the island.

### Statistical analysis

The results of the investigations (clinical forms and environmental forms) were entered into EPIData 3.1 ^®^. Incidence maps per year, and districts were created using Qgis 2.2 software (open source geospatial foundation OSGEO^®^).

## Results

**Fig. 2:**
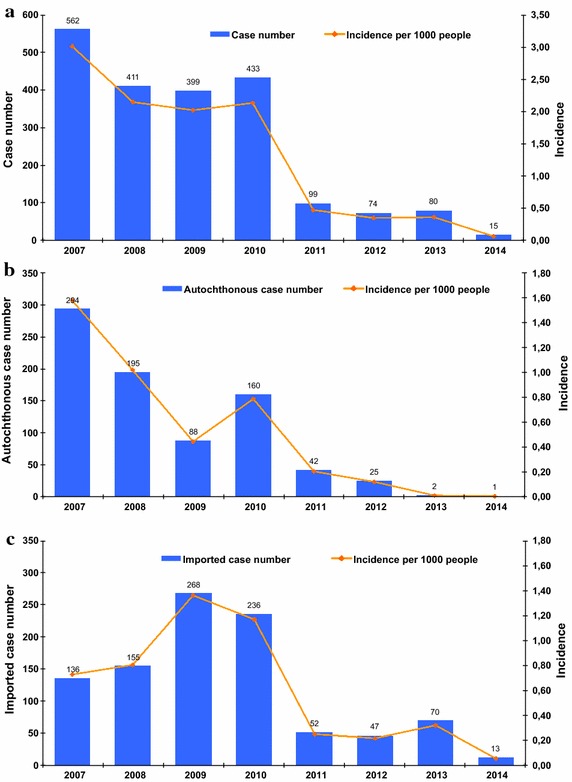
**a** Evolution of the annual incidence of malaria in Mayotte, 2007–2014. **b** Evolution of the annual incidence of autochthonous malaria in Mayotte, 2007–2014. **c** Evolution of the annual incidence of imported malaria in Mayotte, 2007–2014

**Fig. 3:**
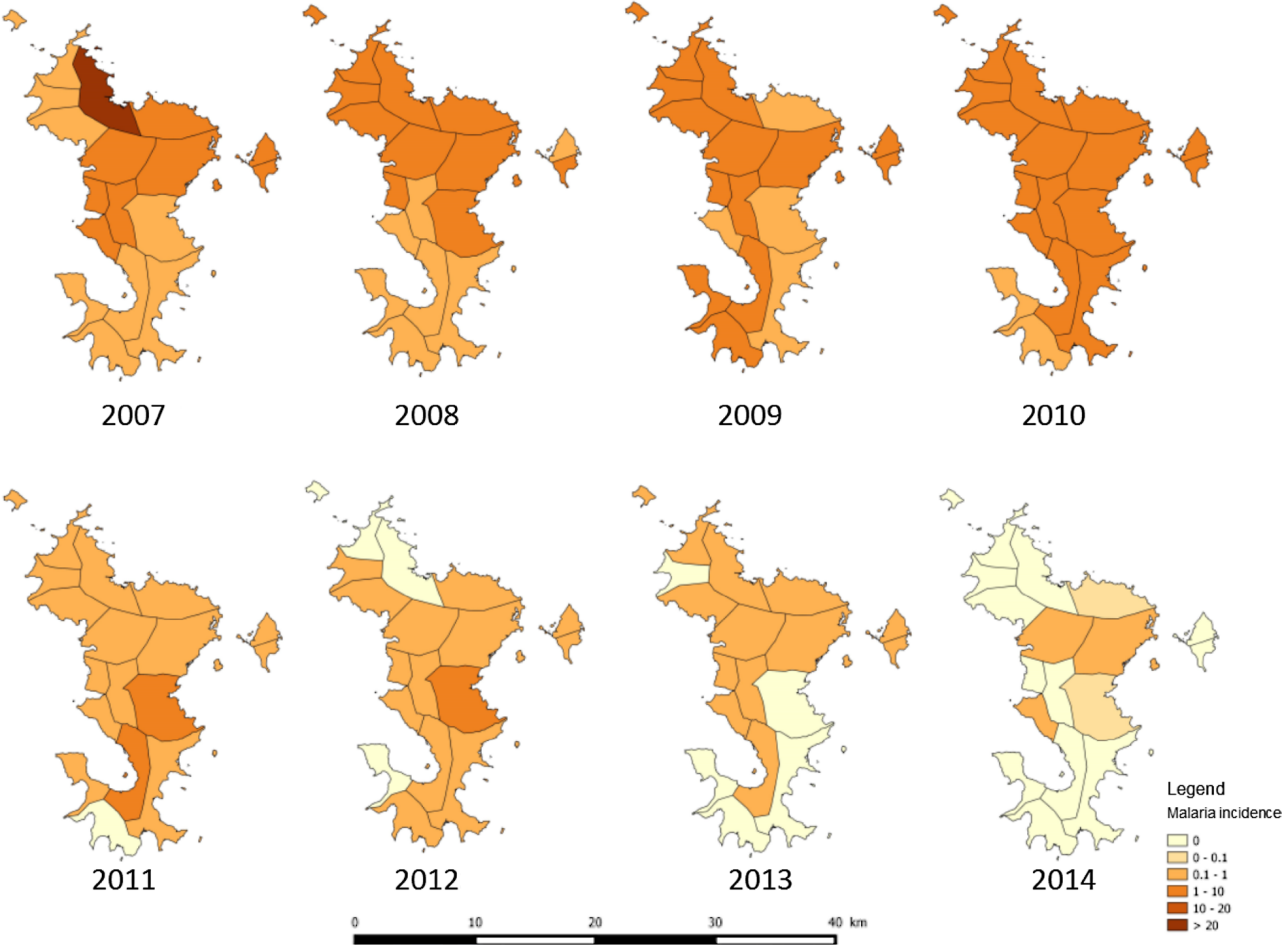
Evolution of the global malaria incidence by district in Mayotte, 2007–2014

From 2007 to 2014, 2,073 cases were reported on Mayotte Island: 977 imported cases, 807 autochthonous cases and 289 unclassified cases (cases of unknown origin). The total malaria API lowered from 3.0 in 2007 to 0.07 per 1,000 inhabitants in 2014 as the autochthonous malaria incidence decreased from 1.6 to 0.004 per 1,000 inhabitants in the same period and in all age groups (Fig. 2). From 2007 to 2010, the large foci of Bandraboua in north Mayotte accounted for 366 cases (46 % of autochthonous cases from 2007 to 2013). Outside the large outbreak of Banbradoua, most of the autochthonous cases were isolated or confined to familial or neighboring clusters around imported cases. Nevertheless, a common point for all of these clusters in recent years was the presence of *An. funestus* as vector. The evolution of the incidence of autochthonous malaria by district is shown in Fig. 3.

After increasing from 2007 to 2010, the number of imported cases has decreased, falling to 13 in 2014. Over the reported period, most of the imported cases came from Comoros (94 %). Hence, the main island, Grand Comoro, accounted for 74 % of the overall cases with an increase from 69 % in 2007 to 84 % in 2013, whereas 16 % of the cases were imported from Anjouan and 2 % of the cases were imported from Moheli. A minority of the cases was imported from Madagascar (4 %) and Africa (approximately 1 %) or other countries. At the same time, the number of cases of unknown origin has also continuously decreased.

*Plasmodium falciparum* was isolated in 93.4 % of the cases over the 8-year period with a downward trend from 2007 to 2009 and a minimum of 88.0 % in 2009 and 2010. In contrast, *P. vivax* represented 2.0 % of the isolates ranging from 0.6 % in 2007 to 5.2 % in 2011; *P. malariae* was found in 4.0 % of the cases, and *P. ovale* was found in 0.4 % of the cases. Over the eight-year period, *P. falciparum, P. ovale, P. vivax and P. malariae* accounted for 93.0, 5.2, 1.5 and 0.3 % of the autochthonous cases, respectively.

**Fig. 4:**
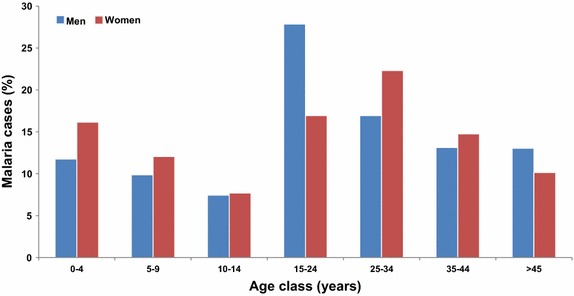
Distribution of malaria cases in % by age class and gender in Mayotte, 2007–2014

Over the period, men represented 62 % of the cases with a minimum of 52 % in 2009 and a maximum of 70 % in 2011. The overall mean age was 23.8 years, with a continuous increase from 2007 to 2014 for both sexes, ranging from 21.6 to 24.8 for women and from 22.9 to 27.7 for men. Thirty-two percent of the cases were less than 15 years of age (of whom 42 % were under five years of age), 24 % of the cases were between 15 and 24 years of age, 32 % of the cases were between 25 and 44 years of age, and 12 % of the cases were over 45 years of age. The age ranges with the most cases were 15 to 24 years for males (28 %) and 25 to 34 years for females (22 %) (Fig. 4). At the same time, malaria has been reported in 40 pregnant women (2 %), including 15 autochthonous cases.

From 2007 to 2014, severe forms represented approximately 11 % of the cases and uncomplicated forms accounted for 89 % of the cases; two deaths were recorded among the imported cases, one 16 years of age in 2007 and one of 2 years of age in 2011. Approximately 19 % of the cases were hospitalized (3 % in an intensive care unit). The hospitalization rate has increased in the last 2 years, to 54 % with 13 % of the cases admitted to an intensive care unit but with no deaths. Since ACT was implemented as first-line malaria therapy in May 2007, 89 % of the cases have been treated with ACT and 10 % of the cases have been treated with quinine (8 % intravenously).

**Fig. 5:**
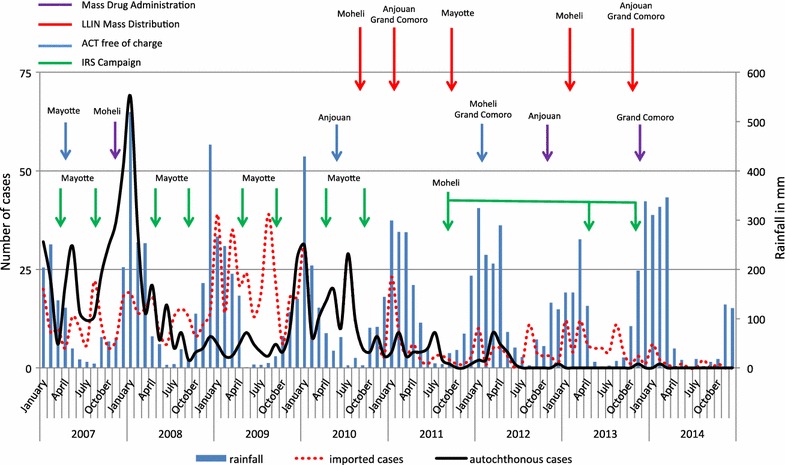
Distribution by month and year of the average rainfall in mm and of the number of autochthonous and imported malaria cases in Mayotte in relation to timing of malaria control interventions in the Comorian Archipelago, 2007–2014

The monthly distribution of both autochthonous and imported cases confirms the continuous transmission throughout the year with a similar distribution pattern each year over the period with two peaks, one in January during the middle of the rainy season and the other one in August during the middle of the dry season, for both autochthonous and imported cases. The rainfall peak of the rainy season was followed by an increase of imported cases but not always by an impact on autochthonous cases. The transmission continued during the dry season with a peak of autochthonous cases in its middle. Each year, a peak of imported cases occurred that could correspond to the end of the holidays on the surrounding islands in August and the beginning of the school year. Nevertheless, the intensity of the transmission also varied according to the year, but those variations were not necessarily explained by climatic changes, except in 2009 as the low autochthonous case number is contemporary with the low rainy rate of this year (Fig. 5).

## Discussion

Because all patients with positive RDT are sampled for a thin/thick smear examination, which is managed in the only hospital laboratory and the single private laboratory, the case number reported might be considered to be exhaustive. Obviously, despite the consultation fee for foreign or clandestine people, it is assumed that no one would be prosecuted if enabled to pay before or after care. Nevertheless, it is possible that the fear of police controls when traveling can limit the recourse to care for some illegal immigrants. Nonetheless, there has clearly been a decrease in malaria transmission in Mayotte since 2007, and the goal of elimination seems more achievable than ever. In 2011, Mayotte entered the elimination phase when *P. falciparum* API passed below one case per 1,000 people at risk [15]. This was confirmed in 2012, 2013 and 2014.

In Mayotte, the fall in autochthonous cases is closely related to the disappearance of the Bandraboua case cluster located in the most incident-heavy district, as shown in Fig. 2. In 2004, the API in Bandraboua was 58.1 per 1,000 inhabitants, whereas the overall annual incidence in Mayotte was 4.6 per 1,000 [16]. Forty percent of cases occurred in Bandraboua during the period 2002–2007, although it represented only 5 % of the population [17]. This rate fell to 20 % in 2008 and to 1 % in 2011; no case has been recorded since 2012. One explanation of this decrease in Bandraboua is the combination of different control measures, including the extensive implementation of LLINs in the villages of the area. Another explanation for the diminution of the autochthonous cases is the reduction in the number of “Tobé” villages around Bandraboua as they were said to be responsible for most of the cluster cases in the area [18]. “Tobé” villages are temporary and precarious villages composed of self-made rustic houses where young men stay for seasonal farming. Often hidden in the woods, these villages were not easily accessible by the vector control department. The main explanation offered for the reduction of these villages is the reinforced measures taken by the French border police as these young men are usually clandestine migrants mostly from Comoros. In these villages, people were probably most exposed to *An. funestus* [5].

The global decrease of malaria transmission in Mayotte is a result of a combination of different measures, such as the integrated active (and passive) surveillance of malaria since 2001, the improved vector control management since 2002 and the change in therapeutic protocol by ACT in 2007. Indeed, regular IRS, the identification and treatment of breeding sites and the distribution of LLIN were all improved [18] as these tools are known to be effective in controlling malaria [19, 20].

The decrease in malaria cases since 2002 seems to be attributed in part to the major decline in autochthonous transmission. In 2002, there were 1,841 malaria cases reported with 194 (11 %) cases imported [16], whereas there were only 15 cases reported with 13 (87 %) cases imported in 2014, one autochthonous and one of unknown origin. In recent years, the implementation of the malaria control program in the surrounding islands has decreased the transmission in the area. In Madagascar, the burden of malaria fell from 90 cases per 1,000 inhabitants in 2001 to below 10 cases per 1,000 inhabitants in 2010, and deaths from malaria in children under 5 years of age decreased from 259 per 1,000 to 72 per 1,000 [21]. However, recent data seem to show a rebound even if those are insufficiently consistent to assess a modification of trends [22]. In the Union of Comoros, a Fast Elimination of Malaria by Source Eradication (FEMSE) and the actions of the National Malaria Control Programme (PNLP) have initiated a major decrease of malaria transmission in the islands of Moheli, Anjouan and Grand Comoro. The global API fell from 109 cases per 1,000 inhabitants in 2011 to 2.8 per 1,000 in 2014 ranging from 0.02 ‰ in Anjouan to 5.47 in Grand Comoro. Moreover, no death occurred in 2014 [23]. This decrease of malaria transmission in the neighbouring countries and particularly in Comoros as they accounted for 94 % of imported malaria cases, contributed to the decrease in imported cases in Mayotte and in the decrease of the local transmission. From 2007 to 2010, the local malaria control activities in Mayotte have conducted to a decrease in the autochthonous cases whereas the number of imported cases has not fallen. Over this period, the malaria control implementations in Moheli have had few impacts as most of imported cases in Mayotte came from Grand Comoro or Anjouan. But from 2010, the number of imported cases has drastically dropped in Mayotte after the beginning of malaria control programmes in those two islands. The evolution of the number of malaria cases in relation to timing of malaria interventions in the Comorian Archipelago from 2007 to 2014 is presented in Fig. 5.

A higher rate of malaria is found among men aged between 15 and 24 years compared to women of the same age class. One cultural explanation of this finding is that young men leave home to build their own hut called “banga”. These precarious dwellings are rarely equipped with LLIN or visited by the vector control department for IRS. These results are consistent with the findings of previous reports on Mayotte [7, 14, 16] and are explained by the particular age distribution in the population, where 53 % of people are under 20 years old.

Results suggest that Mayotte entered the elimination phase of malaria in 2011, according to the new WHO criteria published in 2012 [15]. The WHO criteria and the current situation in Mayotte are presented in Table 1.

**Table 1:** WHO criteria for classifying Mayotte according to the malaria program phase, (source: WHO malaria report [15])

	Pre-elimination	Elimination	Prevention of reintroduction	Mayotte situation
*Malaria situation in areas with most intense transmission*			(1) Recently endemic country with zero local transmission for at least three years or (2) Country on the Register or Supplementary list that has ongoing local transmission*	
Test positivity rate	≤5% among suspected malaria patients (PCD) throughout the year			Yes
API in the district with the highest number of cases/1000 population/year (ACD and PCD)**, average over the last two years	<5 (less than 5 cases/1,000 population)	<1 (less than 1 case/1,000 population)		Yes
Total number of reported malaria cases nationwide		A manageable number e.g. <1000 cases nationwide (local & imported)		Yes
*Case management*			*Imported malaria. Maintain capacity to detect malaria infection and manage clinical disease*	*Imported malaria. Maintain capacity to detect malaria infection and manage clinical disease*
All cases detected in the private sector are microscopically confirmed	National policy being rolled out	Yes	Yes	Yes
All cases detected in the public sector are microscopically confirmed	National policy being rolled out	Yes	Yes	Yes
Nationwide microscopy quality assurance system covers public and private sector	Initiated	Yes	Yes	Yes
Radical treatment with primaquine for *P. vivax*	National policy being updated	National policy fully implemented	Yes	Yes
Treatment with ACT plus single dose of primaquine for *P. falciparum*	National policy being updated	National policy fully implemented	Yes	National policy being updated
*Surveillance*				
Malaria is a notifiable disease nationwide (<24–48 h)	Laws and systems being put in place	Yes	Yes	Yes
Centralized register of cases, foci and vectors	Initiated	Yes	Yes	Yes
Malaria elimination database	Initiated	Yes	Certification process (optional)	Yes
Active case detection in groups at high risk or with poor access to services (“proactive” case detection)	Initiated	Yes	In residual and cleared-up foci, among high risk population groups	Initiated
Case and foci investigation and classification (including “reactive” case detection and entomological investigation)	Initiated	Yes	Yes	Yes

*ACD* active case detection, *PCD* passive case detection

* Ongoing local transmission = 2 consecutive years of local *P. falciparum* transmission, or 3 consecutive years of local *P. vivax* transmission in the same locality or otherwise epidemiologically linked.

** The API has to be evaluated against the diagnostic activity in the risk area (measured as the ABER), low values of ABER in a district raise the possibility that more cases would be found with improved diagnostic efforts.

In 2007, the WHO listed the necessary accomplishments for a malaria elimination programme [24, 25]. In the case of Mayotte, several ones remain to be achieved. First, the whole population, national and foreign, should have easy access to and use of private and/or public health-care facilities, regardless of their citizenship or conditions (refugees, displaced, temporary workers). This implies that dispensaries should continue to provide free or low-price primary medical care for the non-insured population, which comprises mostly clandestine migrants. Second, a malaria elimination programme monitoring committee should be established. Thus, the combined efforts of all stakeholders in the malaria control policy are needed. Third, a programme of joint activities should be established in the Comorian Archipelago, which is probably the most important but most difficult challenge as Mayotte is only 70 km south east of Anjouan, the closest Comoros Island. The Union of Comoros is unstable with some political autonomy for each island. Therefore, Mayotte is an alternative for Comorian people who represented 95 % of the foreigners living in Mayotte in 2007. In Mayotte, clandestine migrants are cumulating malaria risk factors, such as the proximity of dwellings to mosquito breeding sites, living in poorly constructed houses, and having occupations such as forest or animal-related work, which increase their contact with mosquitoes, conferring a high degree of resilience [26].

Additionally, insecticide and drug resistances should be monitored for early detection. Deltamethrin was reported to be efficient on all strains of *An. gambiae* found in Mayotte [4], but recent evidence of pyrethroids resistance was detected in *An. funestus* in Zambezia, Mozambique [27]. Worldwide, insecticide resistance in malaria vectors has been reported in 53 of 65 reporting countries around the world since 2010, most commonly to pyrethroids [22]. In the Comorian Archipelago, malaria control has been hampered by the emergence of *P. falciparum* resistance to chloroquine and to sulfadoxine-pyrimethamine in the early 1980s, as well as resistance of Anopheles mosquitoes to DDT [28, 29]. Genetic studies have shown different resistance patterns between Mayotte and Comoros due to the massive use of chloroquine from 1975 to 2007 and the use of sulfadoxine-pyrimethamine from 2002 to 2007 [17, 29–31].

Therefore, a more rational, regular and efficient chemoresistance surveillance system is urgently needed and should be implemented simultaneously but separately for each island as continual human migrations across the archipelago seem inevitable [17, 32]. Resistance to ACT is also of concern in the Indian Ocean area, and new studies should be conducted to update data and guide first-line treatment policy as ACT has only been used for a few years.

Moreover, a geographic information system-based elimination database should be considered to provide data on vector settings and control interventions as is currently done for human cases [24]. To monitor the effectiveness of the malaria interventions, the surveillance system has to be improved. The notion of “introduced cases” and indigenous cases must be added to the currently used definitions of autochthonous cases and imported cases. Investigations of cases must also be strengthened to significantly decrease the number of unclassified cases. Furthermore, as the number of malaria cases decreases, the vigilance of physicians may also decrease and therefore the prescription of RDTs or blood smears may not be systematically carried out in case of fever. To control this possible trend, the number of RDTs (distributed, used) and the number of slides and thick smears realized should be monitored. This will allow the Annual Blood Examination Rate (ABER) to be calculated, as recommended by the WHO, to detect a decrease in malaria screening and validate the fact that the decrease in cases is due to a real diminution of transmission.

As premunition against malaria lowered in Mayotte with the substantial decline of case incidence [16] and in the context of a still intense population of immigrants from Comoros, acute active surveillance is needed to detect cases and clusters as soon as possible in close conjunction with the vector control department to promptly adjust the malaria control measures and prioritize targeted control strategies, such as active screening around cases. Indeed, in 2011, as the Bandraboua cluster disappeared, two others emerged. Active case detection (ACD) allowed rapid identification of grouped cases, and joint efforts with the vector control department enabled for properly controlling these grouped cases. At the beginning of 2012, a new emerging cluster was identified in the south.

This also implies active case detection for asymptomatic cases targeting at-risk populations such as clandestine migrants owing to the high potential of reemergence in the context of an important migration flow, particularly on an island like Mayotte [33, 34]. To succeed in ACD, the information and involvement of the different communities in Mayotte is necessary [35–37]. In fact, a concerted information, education and communication strategy is crucial for adherence to intervention measures in all communities. Selected volunteers from Comoros should also be used to enroll the clandestine population without stigmatization, which could generate negative social impact and, therefore, be counterproductive for an elimination programme [38].

Although malaria transmission can be controlled and suppressed by effective measures, the resurgence spectrum needs to be considered. A recent systematic review [39] reported that almost all resurgence events between the 1930s and 2000s were at least partially attributed to the weakening of malaria control programmes and resource restrictions (57 %). In the case of Mayotte, past failure of malaria elimination raises the question of the minimal geographic isolation level [31] and the optimal size of the intervention area required for malaria elimination success.

Sustained efforts will be required to prevent the reemergence of malaria from Mayotte. Lowering malaria transmission from the three other islands is part of the challenge and will require more potent tools and interventions and stronger health systems than those available today.

## Conclusions

Worldwide strategies have suggested the efficacy of combined interventions to control malaria. Vector control measures, such as IRS, LLINs, active surveillance and case management, including definitive diagnosis and effective treatment with ACT, are essential tools.

Mayotte has entered the elimination stage. However. some goals remain to be accomplished before a program re-orientation toward malaria elimination is contemplated and a community participation programme should be discussed. Elimination of malaria requires a re-orientation of control activity, moving away from population-based interventions to interventions based on a programme of effective surveillance and response.

*Plasmodium falciparum* transmission is a global health problem in Africa, even on an island, requiring a global strategy with regional targets and approaches tailored to what can be achieved within defined intervention periods. Thus, the elimination programme of Mayotte is closely related to the PNLP of the Union of Comoros that was recently intensified in 2010 with the Roll Back Malaria Partnership. In this way, a regional management policy is crucial as control measures could be targeted and based on a regional surveillance-response system rather than isolated.
